# Germline truncating-mutations in *BRCA1* and *MSH6* in a patient with early onset endometrial cancer

**DOI:** 10.1186/1471-2407-12-531

**Published:** 2012-11-20

**Authors:** Karin Kast, Teresa M Neuhann, Heike Görgens, Kerstin Becker, Katja Keller, Barbara Klink, Daniela Aust, Wolfgang Distler, Evelin Schröck, Hans K Schackert

**Affiliations:** 1 Department of Gynecology and Obstetrics, University Hospital Carl Gustav Carus, Technische Universität Dresden, Dresden, Germany; 2 Institute for Clinical Genetics, Technische Universität Dresden, Dresden, Germany; 3 Medical Genetic Center, Munich, Germany; 4 Department of Surgical Research, University Hospital Carl Gustav Carus, Technische Universität Dresden, Dresden, Germany; 5 Institute of Pathology, University Hospital Carl Gustav Carus, Technische Universität Dresden, Dresden, Germany

## Abstract

**Background:**

Hereditary Breast and Ovarian Cancer Syndrome (HBOCS) and Hereditary Non-Polyposis Colorectal Cancer Syndrome (HNPCC, Lynch Syndrome) are two tumor predisposition syndromes responsible for the majority of hereditary breast and colorectal cancers. Carriers of both germline mutations in breast cancer genes *BRCA1* or *BRCA2* and in mismatch repair (MMR) genes *MLH1, MSH2, MSH6* or *PMS2* are very rare*.*

**Case presentation:**

We identified germline mutations in *BRCA1* and in *MSH6* in a patient with increased risk for HBOC diagnosed with endometrial cancer at the age of 46 years.

**Conclusions:**

Although carriers of mutations in both MMR and BRCA genes are rare in Caucasian populations and anamnestical and histopathological findings may guide clinicians to identify these families, both syndromes can only be diagnosed through a complete gene analysis of the respective genes.

## Background

Hereditary Breast and Ovarian Cancer Syndrome (HBOCS) is an autosomal dominantly inherited disease caused by mutations in *BRCA1* or *BRCA2* and characterized by young age of onset, synchronous or metachronous disease, and a family history of first and second degree relatives with breast and/or ovarian cancer. Depending on the affected gene, the estimated lifetime risks range from 46-85% for breast and 11-53% for ovarian cancer [1,2].

Lynch syndrome (hereditary non-polyposis colorectal cancer, HNPCC), is also an autosomal dominant tumor predisposition caused by germline mutations in DNA mismatch repair (MMR) genes *MLH1, MSH2, MSH6* or *PMS2* with a life-time risk for colorectal cancer (CRC) of up to 80% and considerably increased risk of developing a broad spectrum of extracolonic malignancies including, among others, endometrial cancer (EC), stomach cancer and ovarian cancer [3,4]. In females, the cumulative risk for EC is 17-57% and almost equally high for CRC [5-8]. In *MSH6* gene mutation carriers, CRC and ovarian cancer risks are lower [9]. However, *MSH6* is only affected in a small fraction of all MMR gene mutation carriers [6,8].

Little is known about the phenotype of families with double mutations causing both HNPCC and HBOCS. Therefore, we report on a female index patient in a family fulfilling the criteria for HBOCS which developed endometrial cancer at age 46 and was identified as a double heterozygous germline mutation carrier.

## Case presentation

**Figure 1:**
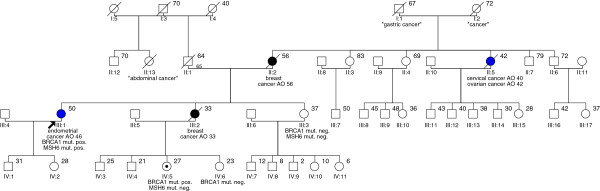
**Pedigree.** The black arrow indicates the index patient and the black dot the unaffected female carrier of the familial *BRCA1* mutation. Documentation was not available for the “gastric cancer” reported in I:1. Abbreviations: AO- age of onset, mut. ‐ mutation, pos. ‐ positive, neg. ‐ negative.

The index patient of the family (individual III:1) was diagnosed with early stage endometrioid endometrial cancer at the age of 46 years, thus almost meeting the original Bethesda Guideline 4 [10]. She was referred to our Center for Hereditary Breast and Ovarian Cancer because of the early demise of her sister (III:2) due to triple negative breast cancer at the age of 33 years. Likewise, her mother (II:2) suffered from triple negative breast cancer at the age of 56. Her maternal aunt (II:5) had papillary-serous ovarian cancer at the age of 43 and additionally had been diagnosed with cervical squamous cell carcinoma at the age of 40. Moreover, the patient reported that her maternal grandfather I:1 had died of gastric cancer at the age of 67 years (no histologic or pathologic report were available). Four further maternal aunts and uncles and all of their children had not been reported for malignant diseases. One case of abdominal cancer of unknown origin (II:13) was reported in the paternal pedigree (Figure 1). All family members with breast or ovarian cancer had died. After counseling of the index patient and obtaining informed consent, analysis of the breast cancer genes *BRCA1* and *BRCA2* and testing for HNPCC was performed.

DNA-analysis of *BRCA* genes *1* and *2* was carried out by pre-screening of DNA extracted from lymphocytes of the index patient (III:1) with DHPLC (Denaturing High Performance Liquid Chromatography) using the WAVE® System 2100 (Transgenomic Ltd, United Kingdom) as described elsewhere [11]. Sanger sequencing of suspect fragments revealed the pathogenic *BRCA1* mutation c.213-12A>G, p.Arg71SerfsX21. This nucleotide change is predicted to form a cryptic splice site resulting in the addition of 11 nucleotides to the *BRCA1* transcript and a truncated protein [12]. Immunohistochemical staining (IHC) of MMR proteins MLH1*,* MSH2*,* MSH6 and PMS2 was carried out on formalin-fixed, paraffin-embedded tumor sections of the endometrial cancer of individual III:1 as described elsewhere [13]. Loss of expression of MSH6 was identified. Consequently, sequencing of the *MSH6* gene was performed using primers and conditions described elsewhere [14], which revealed the pathogenic mutation c.515_516insT, p.Ile172fsX10.

The index patient (III:1) was thus identified as a carrier of pathogenic germline mutations in both *BRCA1* and *MSH6*. At the time of counseling she was 51 years of age. The endometrial cancer had been diagnosed at stage pT1a pNx G3 (FIGO IA) and was treated with hysterectomy, bilateral adnexectomy and resection of the upper proximal part of the vagina. Although facing a lifelong estimated risk for breast cancer of up to 80% [2] she decided against preventive surgery. In addition to breast cancer surveillance, screening exams for HNPCC-associated tumors according to the German HNPCC Consortium [15] have been regularly performed in the last three years without pathological findings.

Her sister (III:3) and her nieces (IV:5 and IV:6) underwent predictive testing for the *BRCA1* mutation of which IV:5 was identified to be a carrier. The sister (III:3) of the index patient and one niece (IV:5) were tested for the familial *MSH6* mutation and none of them carries the mutation. At the time of writing this article, no further predictive testings have been carried out in other family members.

## Conclusions

To date, only two families with co-occurrence of a MMR and *BRCA1/BRCA2* mutation have been reported [16,17]. Thiffault et al. described two protein truncating mutations in *MSH2* and *BRCA2* identified in one female individual with breast cancer at the age of 32 and colon polyps at the age of 40. The patient’s father (who had died at the age of 36 years) was an obligate carrier of both mutations and had CRC at 32 years. The female patient described by Borg et al. carried two *MLH1* missense mutations (one of which segregating with familial, Lynch syndrome associated tumors) plus a *BRCA1* mutation, being diagnosed with breast cancer at the age of 35 years. The reported tumors in these patients and the hereby reported case do not exceed the spectrum of HBOCS/HNPCC associated malignancies and are within the expected age range of disease onset for carriers of a germline mutation in the respective MMR or BRCA genes alone.

In our family, the only Lynch syndrome associated malignancy was the EC in the index patient. Although three other cancers in both family branches (I:1, I:2, II:13) have been described, no proof of the tumor origin and/or pathology findings were available. It cannot be determined from the pedigree whether the *MSH6* mutation originates from the maternal or paternal side. Since it is unlikely that the *MSH6* mutation represents a *de novo* mutation, the low frequency of Lynch Syndrome associated cancers in both branches of the family may be due to reduced penetrance of *MSH6* mutations [8,9,18]. So far, only two members of the family consented for predictive testing for the *MSH6* mutation, none of which carried the mutation.

No consensus exists regarding the association of breast cancer with Lynch Syndrome. Although MSI has been described in most breast cancers of HNPCC kindreds, the risk for breast cancer is not elevated in most families [5,19-23]. However, a recent prospective study revealed an almost four-fold increase of breast cancer incidence in carriers of a mutation in the HNPCC-genes [24]. Moreover, Scott et al. reported on a significant overrepresentation of breast cancers in *MLH1* mutation carriers [25].

Triple negativity has been shown to be strongly associated with mutation carrier status of *BRCA1*[26]. Thus, two triple negative breast cancers and the presence of an ovarian cancer strongly suggested a *BRCA1*-associated disease in this family (Figure 1). Furthermore, a slightly increased risk for uterine and cervical cancer as well as colorectal cancer has also been reported for *BRCA1* mutation carriers [27]. The uterine cancer of III:1 could therefore be misinterpreted as caused by the *BRCA1* germline mutation*.* Nevertheless, HBOCS-associated uterine cancers tend to be of serous papillary type, whereas HNPCC-associated uterine cancers typically are of endometrial type, as in our case [28]. Proven by loss of expression of MSH6 the EC in patient III:1 is due to the germline *MSH6* mutation. Additionally, the above mentioned higher rate of cervical cancers may be due to the interaction of the HPV oncogenes E6 and E7 with *BRCA1*, which has been shown to render the cervix more susceptible to cancer [29].

The incidence of ovarian cancer in HBOCS is higher than in Lynch Syndrome. Additionally, *BRCA1*-associated ovarian cancers often show high grade serous histology, whereas HNPCC-associated ovarian cancers often display endometrioid histology [23,30]. Therefore, the ovarian cancer of individual II:5 was most probably *BRCA1-*associated.

It can be assumed that revised Bethesda Guidelines recommendations for the identification of colorectal tumors that should be tested for MSI, sometimes fail to identify individuals with HNPCC. To avoid non-identification of HNPCC-patients it might be reasonable to screen both, colorectal and endometrial cancer tumor specimens for MMR-defect. Interestingly, this topic has been discussed at least for colorectal cancer at the HNPCC workshop conducted by the NCI in Bethesda, MD in 2002. Although participants voted to keep less than 60 years of age in the guideline 3 there was no consensus on whether to include the age criteria [4].

Data from the Robert Koch Institute German Cancer Registry ( http://www.rki.de) indicate that the incidence of HNPCC associated cancers such as colorectal, small bowel, urinary tract, and endometrial cancer is about 6% in the German population. Assuming that 5% of these tumors are caused by MMR gene mutations with a penetrance of 80%, the allele frequency in the German population is 0.38% [31]. Incidence of breast cancer is 9.53% in the German female population. On the assumption that 5% of these tumors are caused by *BRCA1* or *BRCA2* mutations with a penetrance of 85% and that the allele frequency is similar in the male population, the allele frequency in the whole German population is 0.6% [32,33]. Therefore, the probability to be a carrier of both mutations in breast cancer genes and MMR genes is 1/167 × 1/260 × 1/4 = 1/174.800, which sums up to 470 carriers of both alleles in the German population. Since allele frequencies are similar in Western populations the frequency of carriers of both alleles may be up to 5.7 per one million individuals.

Although carriers of mutations in both MMR and BRCA genes are rare in Caucasian populations, anamnestical and histopathological findings may guide clinicians to identify these families. Firstly, meeting both Bethesda Guidelines and HBOCS Criteria may raise suspicion of both syndromes in one family. Secondly, this may be substantiated by specific histological findings in available tumors, such as triple negative breast cancers (*BRCA1*-associated), high grade papillary serous ovarian cancer (*BRCA1*- and *2*-associated), endometrioid cancer of the uterus (MMR-gene-associated) and microsatellite instability (MMR-gene-associated). Finally, both syndromes can only be diagnosed through a complete gene analysis of the respective genes.

## Consent

Written informed consent was obtained from the index patient III:1 and from relative IV:5 for publication of this case report and any accompanying images.

## Misc

Karin Kast and Teresa M Neuhann contributed equally to this work.

## Competing interests

The authors declare that they have no competing interests.

## Authors' contributions

Karin Kast worked on the concept and design of the study and wrote the manuscript, also contributing to the acquisition, analysis and interpretation of the data. Teresa Neuhann recruited the family, initiated the genetic diagnostics of the BRCA and HNPCC genes, suggested the publication of the case and contributed to the writing of the manuscript. Katja Keller, Kerstin Becker and Barbara Klink were involved in data acquisition and communication with the patients. Daniela Aust and Heike Görgens were fundamental for the analysis and interpretation of the data. Evelin Schröck contributed to the final manuscript and oversaw the genetic counseling. Wolfgang Distler approved the manuscript to be published. Hans K. Schackert advised and contributed to the concept, design and writing the manuscript, as well as to the analysis and interpretation of data. All authors read and approved the final manuscript.

## Pre-publication history

The pre-publication history for this paper can be accessed here:

http://www.biomedcentral.com/1471-2407/12/531/prepub
